# Silicon-Based Biosensors: A Critical Review of Silicon’s Role in Enhancing Biosensing Performance

**DOI:** 10.3390/bios15020119

**Published:** 2025-02-18

**Authors:** Waqar Muhammad, Jaeyoon Song, Sehyeon Kim, Falguni Ahmed, Eunseo Cho, Huiseop Lee, Jinsik Kim

**Affiliations:** Department of Biomedical Engineering, College of Life Science and Biotechnology, Dongguk University, Seoul 04620, Republic of Korea; waqar6414@dgu.ac.kr (W.M.); thdwodbs95@dongguk.edu (J.S.); kshsarah@dongguk.edu (S.K.); falguni.dg@dgu.ac.kr (F.A.); 2021111733@dgu.ac.kr (E.C.); 2018111717@dgu.ac.kr (H.L.)

**Keywords:** silicon, CMOS, biosensors, miniaturization, sensitivity, specificity

## Abstract

This review into recent advancements in silicon-based technology, with a particular emphasis on the biomedical applications of silicon sensors. Owing to their diminutive size, high sensitivity, and intrinsic compatibility with electronic systems, silicon-based sensors have found widespread utilization across healthcare, industrial, and environmental monitoring domains. In the realm of biomedical sensing, silicon has demonstrated significant potential to enhance human health outcomes while simultaneously driving progress in microfabrication techniques for multifunctional device development. The review systematically examines the versatile roles of silicon in the fabrication of electrodes, sensing channels, and substrates. Silicon electrodes are widely used in electrochemical biosensors for glucose monitoring and neural activity recording, while sensing channels in field-effect transistor biosensors enable the detection of cancer biomarkers and small molecules. Porous silicon substrates are applied in optical biosensors for label-free protein and pathogen detection. Key challenges in this field, including the interaction of silicon with biomolecules, the economic barriers to miniaturization, and issues related to signal stability, are critically analyzed. Proposed strategies to address these challenges and improve sensor functionality and reliability are also discussed. Furthermore, the article explores emerging developments in silicon-based biosensors, particularly their integration into wearable technologies. The pivotal role of artificial intelligence (AI) in enhancing the performance, functionality, and real-time capabilities of these sensors is also highlighted. This review provides a comprehensive overview of the current state, challenges, and future directions in the field of silicon-based biomedical sensing technologies.

## 1. Introduction

The integration of sensor technology into modern electronics has significantly transformed various fields, enabling precise and continuous monitoring in healthcare, environmental surveillance, and industrial applications. Among the available sensor technologies, Complementary Metal–Oxide–Semiconductor (CMOS)-based sensors have played a pivotal role due to their scalability, energy efficiency, and compatibility with existing electronic systems [1,2]. Although CMOS technology was developed in the mid-20th century, its application in miniature sensor systems has expanded dramatically since the 1980s [3,4,5]. Over time, silicon has emerged as the dominant material for fabricating these sensors, owing to its exceptional electrical properties, cost-effectiveness, and ease of integration with microfabrication techniques [6,7]. Also, silicon-based biosensors have garnered significant attention due to their high sensitivity, stability, and ability to detect biological markers with precision [8,9]. These sensors leverage silicon’s inherent properties, including its excellent charge carrier mobility, compatibility with miniaturized circuitry, and ability to function under diverse environmental conditions [10,11]. Their application spans multiple domains, from diagnostic tools to real-time monitoring of biological signals, offering novel solutions in medical research and personalized healthcare [12,13]. One of the key advantages of silicon biosensors is their adaptability, which allows for modifications in sensor architecture to optimize performance for specific biological targets. This adaptability extends to various sensor configurations, including field-effect transistors (FETs) [14,15], capacitive sensors [16,17], optical biosensors [18,19], and electrochemical detection platforms [20,21].

While silicon-based biosensors offer numerous advantages in biomedical applications, their performance and functionality are largely dependent on precise fabrication techniques. However, achieving optimal efficiency and reliability requires advanced microfabrication processes. Techniques such as photolithography defines intricate patterns on silicon wafers using photoresists and UV exposure, followed by etching (wet or dry) to shape sensor structures. Thermal oxidation creates insulating silicon dioxide layers crucial for FET-based biosensors, while thin-film deposition (sputtering, CVD, ALD) enables electrode and surface modifications and spin-coating ensures uniform polymeric or biochemical coatings for biofunctionalization. These processes collectively enhance sensitivity, stability, and selectivity, enable the creation of nanoscale sensor architectures, enhancing detection accuracy and stability [22,23,24].

In comparison with other materials, silicon biosensors excel in electrical conductivity, mechanical robustness, and stability. While polymer-based sensors offer flexibility and cost-effectiveness [25], their performance characteristics are limited. Indium Phosphide (InP) biosensors provide high carrier mobility for photonic applications [26], but are more complex and expensive to fabricate. Silicon nitride (Si_3_N_4_) offers superior chemical resistance and optical properties [27], but falls short in electrical performance compared to silicon. Despite these alternatives, silicon remains the material of choice due to its CMOS compatibility, high integration potential, and established fabrication processes. Recent advancements in nanotechnology have further refined silicon biosensors, enabling the development of nanowires, microcantilevers, and other nanostructures that enhance signal transduction and sensitivity [8,12,28]. These innovations have significantly advanced biosensing capabilities, allowing for highly selective and rapid detection of biomarkers such as proteins, nucleic acids, and small metabolites [29,30,31]. Beyond conventional biomedical applications, silicon biosensors are being explored for their role in advanced diagnostics, therapeutic monitoring, and drug delivery systems [32,33]. In disease diagnostics, these sensors facilitate the detection of biomarkers associated with conditions such as cancer, infectious diseases, and neurodegenerative disorders [34,35]. Notably, silicon biosensors with microfluidic integration enable real-time detection of circulating tumor biomarkers in blood, saliva, and urine. This liquid biopsy approach advances cancer diagnosis by allowing non-invasive, early-stage detection and monitoring of tumor-derived biomarkers [36,37]. Their integration into lab-on-a-chip platforms has revolutionized point-of-care testing, allowing rapid and accurate results without the need for complex laboratory infrastructure. Additionally, silicon biosensors have been extensively utilized to monitor metabolic parameters such as glucose levels, electrolyte concentrations, and enzymatic activity [38,39], as well as physiological parameters including heart rate, blood pressure, and body temperature [40,41]. These sensors, such as silicon nanowires (SiNWs) [42], field-effect transistors [43], and microcantilever-based sensors [44], offer high sensitivity and precision in detecting these parameters, enabling real-time and continuous monitoring.

Despite their numerous advantages, silicon-based biosensors face several challenges that need to be addressed to enhance their clinical applicability. One major limitation is their sensitivity to environmental factors such as temperature fluctuations, humidity, and biofouling, which can affect measurement accuracy [45]. Efforts to mitigate these issues have focused on surface functionalization techniques that improve selectivity and stability while minimizing nonspecific interactions [46]. Another challenge is the complexity of integrating silicon biosensors into biological environments without compromising their functionality. This has led to extensive research on biocompatible coatings, flexible substrates, and hybrid materials that enhance sensor performance while ensuring minimal interference with biological systems [47]. Additionally, the economic and technical challenges associated with large-scale fabrication and miniaturization must be considered. While silicon-based biosensors benefit from well-established semiconductor manufacturing processes, their mass production still requires optimization to reduce costs and improve scalability. Advances in additive manufacturing, microfluidic integration, and alternative deposition methods are being explored to address these issues [48,49]. Furthermore, optimizing signal processing algorithms and wireless data transmission techniques is essential for real-time and remote biosensing applications [50,51]. The future of silicon biosensors lies in their continued refinement and integration with emerging technologies such as artificial intelligence (AI), machine learning, and Internet of Things (IoT) frameworks [52,53]. These advancements will enable automated data analysis, predictive modeling, and enhanced decision-making in clinical and research settings.

**Figure 1:**
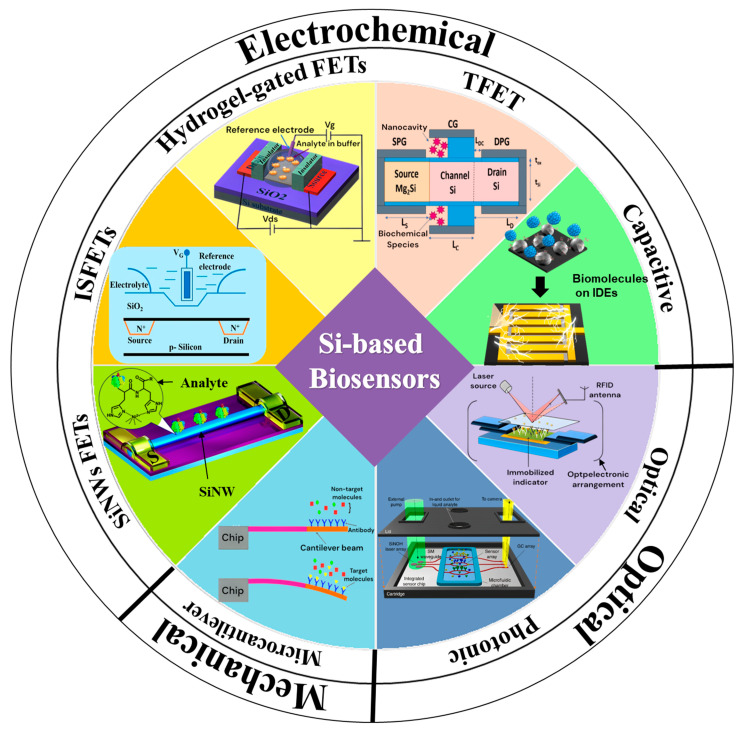
Classification of silicon-based biosensors categorized into three major types: electrochemical, optical, and mechanical biosensors. Each quadrant highlights key sensing mechanisms, including SiNWs FETs, Copyright 2016, Royal Society of Chemistry [54]. ISFETs, reproduced from [55] under Creative Commons Attribution License, 2022. Hydrogel-gated FETs, Copyright 2018, Elsevier [56]. TFET, Copyright 2024, Elsevier [57]. Capacitive, Copyright 2022, Elsevier [58]. Optical, reproduced from [59] under Creative Commons Attribution License, 2011. Photonic, reproduced from [60] under Creative Commons Attribution License, 2021. Microcantilever, Copyright 2024, Springer Nature [61].

Although significant progress has been made in the development and deployment of silicon-based biosensors, gaps remain in fully understanding their long-term biological interactions and optimizing their use for complex biomedical applications. These challenges include a limited understanding of how silicon biosensors interact with biological tissues over extended periods and the difficulties in maintaining sensor stability and accuracy in dynamic environments. This review provides a comprehensive analysis of recent advancements in silicon-based biosensors, categorized in Figure 1 according to their classification as electrochemical, optical, and mechanical biosensors. It systematically explores their fabrication techniques, fundamental operational principles, and biomedical applications. Additionally, it highlights critical challenges associated with their implementation and discusses emerging strategies to enhance performance, scalability, and integration into next-generation healthcare technologies. By providing an in-depth analysis of the existing literature, this work aims to bridge the gap between research advancements and practical implementations, paving the way for future innovations in silicon-based biosensing systems.

## 2. Development Process of Different Silicon-Based Biosensors

### 2.1. Silicon-Based Electrochemical Biosensors

Electrochemical biosensors facilitate the detection of biomolecular interactions by transducing biochemical signals into measurable electrical responses, such as changes in current, voltage, or impedance [37,40]. These sensors exhibit high sensitivity, rapid response times, and miniaturization potential, making them highly suitable for biomedical diagnostics. Silicon-based electrochemical biosensors, including SiNW FETs, ISFETs, and capacitive biosensors, exploit silicon’s superior electrical properties and compatibility with advanced microfabrication techniques to achieve precise and real-time analyte detection.

#### 2.1.1. SiNW FET Biosensors

**Figure 2:**
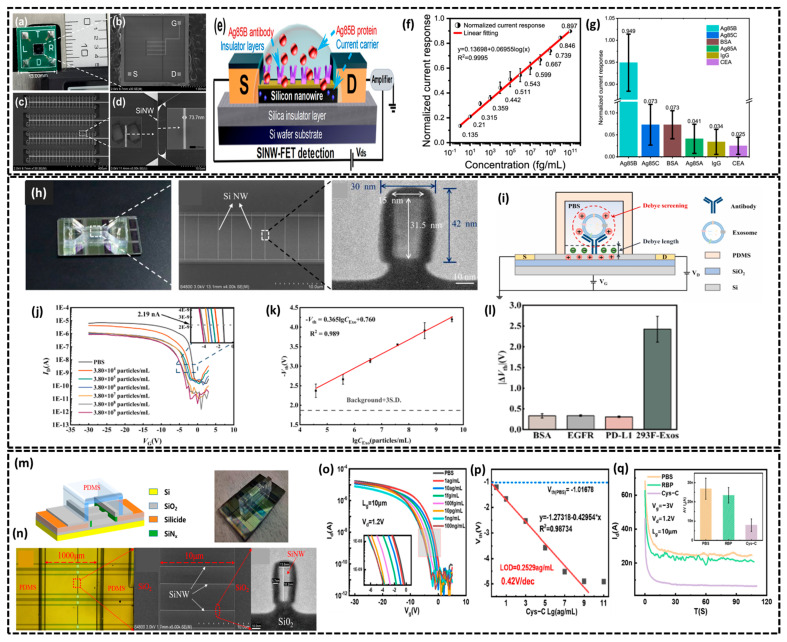
(**a**) Image showing the packaged SiNW-FET device. (**b**) Scanning electron microscope (SEM) image of the SiNW-FET device. (**c**) SEM view of a region with an array of silicon nanowires. (**d**) SEM image of a silicon nanowire from the schematic diagram of the SiNW-FET device. (**e**) Schematic illustration of the biosensor detection mechanism of the SiNW-FET. (**f**) Normalized current response with the Ag85B concentration gradient. (**g**) Normalized current response of the SiNW-FET to 10 ng/mL, demonstrating the biosensor’s specificity in detecting six different proteins. (**a**–**g**) reproduced with permission from the American Chemical Society, Copyright [2021]. (**h**) (Left to right) Photograph of the packaged SiNW Bio-FET device, SEM image of the Si-NW array, and TEM image of the cross-section of the SiNW. (**i**) Schematic diagram of the SiNW Bio-FET for exosomes detection. (**j**) I_D_-V_G_ curves of the anti-CD81 functionalized SiNW Bio-FET after incubation with different concentrations of exosomes derived from 293F cells. (**k**) The Vth of Si-NW Bio-FET to 293F-derived exosomes at a series of concentrations. (**l**) The Vth of the SiNW Bio-FET in response to 293F-derived exosomes at various concentrations. (**h**–**l**) reproduced with permission from Elsevier, Copyright [2024]. (**m**) Schematic of the SiNW biosensor featuring a microfluidic channel, (**n**) (left to right) photograph of the SiNW biosensor, optical microscope image showing the partially amplified SiNW biosensor, SEM image of the SiNW array within the open channel, TEM image of the SiNW. (**o**) Transfer characteristics of the SiNW FET biosensor in response to different concentrations of Cys-C (the red box highlights the shift in the curve near the sub-threshold region). (**p**) The Vth of the SiNW FET biosensor in a varying Cys-C concentrations. (**q**) The modified antibody SiNW FET biosensor’s electrical performance in detecting various biomarkers of early kidney failure. (**m**–**q**) reproduced from [67] under Creative Commons Attribution License, 2023.

Silicon nanowire field-effect transistors (SiNW FETs) have garnered significant interest in biosensing platforms due to their unique characteristics, including ultra-high sensitivity, scalability for miniaturization, and a high surface-to-volume ratio, which enables precise and selective biomolecular detection [62,63]. This sensitivity is leveraged to convert molecular interactions, such as those with proteins, DNA or RNA into electrical signals which is critical in wearable bioelectronics that require continuous monitoring of biological markers [64]. SiNW FET biosensors achieve this by functionalizing the nanowire surface with biomolecules engineered to selectively bind specific target biomarkers. When a target binds to the SiNW surface, it modulates the device’s conductance through changes in the internal charge density leading to highly specific and sensitive biosensing. Despite these advancements, challenges remain such as improving device stability and optimizing integration into flexible, low-power wearable formats. However, recent innovations in the SiNW FET design are gradually addressing these issues, paving the way for robust SiNW biosensors. In this regard, Jinbiao et al. fabricated a highly sensitive CMOS-compatible p-type SiNW-FET biosensor, optimized for Mycobacterium tuberculosis (MTB) detection using antigen 85B (Ag85B) as a biomarker [65]. A highly responsive array of 160 SiNWs was created on a (111) silicon-on-insulator (SOI) wafer using “top down” microfabrication techniques, as shown in Figure 2a–d. The SiNW-FET biosensor was designed with an inverted triangle-shaped silicon nanowire structure to maximize the surface-to-volume and depletion ratios, enhancing sensor sensitivity. As shown in Figure 2a–c, the sensor is packaged into a compact 13 mm × 13 mm unit on a PCB board, including 160 well-arranged SiNWs arrays with gold contacts. An SEM image (Figure 2d) confirmed a surface oxide layer with a narrow width of 73.7 nm, contributing to favorable transport properties due to the SiNW’s high surface-to-volume ratio. The biofunctionalization of the SiNW surface involved plasma treatment (80 W, 150 s) to generate hydroxyl groups, followed by immersion in a 2% APTES ethanol solution overnight to introduce amino groups. Glutaraldehyde (25%) was used as a linker for 2.5 h, enabling covalent attachment of anti-Ag85B antibodies (133 μg/mL). Nonspecific binding sites were blocked with 100 mM ethanolamine. This functionalization ensured specific and efficient antibody immobilization, critical for sensitive biosensing. Displayed in Figure 2e is the detection principle of the SiNW FET biosensor, which involves immobilizing the MTB-specific bioreceptor, anti-Ag85B antibody onto the SiNW, which serves as the gate of the FET. The sensor converts the biological interaction between the antibody and target biomolecule Ag85B into a measurable change in the FET’s current. When an antibody (Ab) with charge *X1* interacts with an antigen (Ag) carrying charge *X2*, an antibody–antigen complex with total charge *X3* is formed. This reaction can be represented as follows:

Ab*^X1^ +* Ag*^X2^* Ab ⇔ [AbAg]*^X3^* (1)

The change in charge from *X1* to *X3* is measurable through the immuno-FET, which detects this charge difference as a change in the current. Moreover, this SiNW FET-based immune sensor detects biological interactions by changes in current. Figure 2f displayed a normalized current showing a linear relationship with the logarithmic value of the Ag85B concentration. The current change value increases linearly as the Ag85B concentration rises across a range of 12 dilution gradients (1 fg/mL to 100 μg/mL) with a high sensitivity of 5.96 nA/log (fg/mL). Thess biosensor achieves an ultra-low limit of detection (LOD) of 0.01 fg/mL while MTB detection completes within 30 s, demonstrating its stability and rapid response time. Finally, the SiNW-FET biosensor demonstrated high specificity for Ag85B with a strong signal change of 0.95, while responses to other tested proteins (BSA, IgG, CEA, Ag85A, and Ag85C) remained below 0.1, indicating minimal unspecific binding as illustrated in Figure 2g. Also, Qin et al. developed a CD81 antibody-modified p-doped SiNW FET biosensor using the CMOS-compatible microfabrication technique for the detection of 293F-derived exosomes [66]. The integrated Si-NW Bio-FET comprises a Si-NW FET device and a PDMS microfluidic channel (15 mm × 500 μm × 100 μm) assembled after oxygen plasma treatment, shown in Figure 2h. The sensor includes 85 SiNWs (individual SiNW is 10 μm long) covered with SiO_2_ with a width of 30 nm and height of 42 nm, enhancing detection capabilities (Figure 2h). The detection principle of the SiNW Bio-FET, illustrated in Figure 2i, involves capturing exosomes through antibodies immobilized on the SiNW Bio-FET. Moreover, the Debye screening effect was investigated by varying electrolyte concentrations of phosphate-buffered saline (PBS), which significantly influenced the sensing limit of the biosensor. The Debye length (*λ_D_*) is given by the following:

(2) $$\lambda_{D}=\sqrt{\frac{\varepsilon_{r}\varepsilon_{0}kT}{2N_{A}q^{2}I}}$$

Here, *ε_r_* represents the relative permittivity of the electrolyte, *ε*_0_ is the vacuum permittivity, *k* is Boltzmann’s constant, *T* is the temperature, *N_A_* is Avogadro’s number, *q* is the elementary charge, and *I* denote the ionic strength of the buffer. It is evident that *λ_D_*, the Debye length decreases as the ionic strength of the solution increases. The biosensor exhibited the maximum electrical response at 0.01 × PBS with an LOD of 1078 particle/mL. Furthermore, the sensitivity of the Si-NW Bio-FET was evaluated using a series of 293F-derived exosome samples at varying concentrations. The I_D_-V_G_ curve in Figure 2j shows a negative shift in the threshold voltage (Vth) as the concentration of exosome samples increased the Vth decreased as the exosome concentration increased showing a linear relationship with the logarithmic value of the concentration (lg_CEXO_) within the range of 3.80 × 10^4^ to 3.80 × 10^9^ particles/mL (Figure 2k). The SiNW Bio-FET specificity (Figure 2l) was determined by modification with anti-CD81 and tested with BSA, EGFR, PD-L1 (each 5 μg/mL) and 293F-derived exosomes (3.80 × 10^9^ particles/mL) with 0.01× PBS as a control. The |ΔV_th_| value for 293F exosomes (2.424 V) was at least seven times higher than for nonspecific targets confirming high specificity. Later, Hu et al. developed an ultrasensitive silicon nanowire biosensor for detecting the early kidney failure biomarker, Cystatin C [67]. The biosensor schematic in Figure 2m depicts the wafer-scale SiNW FET biosensor featuring a 13.5 nm SiNW fabricated using spacer image transfer (SIT) processes. As shown in the actual biosensor image in Figure 2m, the bio-FET device is integrated with a microfluidic PDMS channel (8 × 1 mm × 100 µm) facilitating precise and efficient delivery of antibodies and target molecules to the SiNW surface significantly enhancing detection stability. Figure 2n includes an SEM image of the uniform SiNW arrays and a TEM image showing the final SiNW dimensions of 13.5 × 32.2 nm achieved through the SIT process. Following the fabrication of the biosensor, the sensitivity to Cys-C detection and electrical responses were measured by exposing the SiNW FET surface (modified with anti-Cys C) to varying concentrations of samples ranging from 1 ag/mL to 10 μg/mL. The transfer curves of the biosensor at different concentrations shown in Figure 2o exhibited a negative shift. This shift resulted from the binding of positively charged Cys-C proteins to the SiNW surface, reducing hole concentration in the SiNWs, which increased resistance and decreased current flow. Moreover, the LOD (0.2529 ag/mL) and high sensitivity to Vth (0.42 V/dec) was calculated through a linear fitting curve (Figure 2p) between Vth and the logarithm of the Cyc-C concentration. The specificity of the SiNW bio-FET was assessed by measuring various early kidney failure biomarkers including Cys-C and RBP at a concentration of 1 ng/mL. The results shown in Figure 2q indicate that the bio-FET modified with antibodies exhibited a significant decrease in signal (from 27 nA to 8 nA), exhibiting the excellent specificity of the biosensor. In summary, the SiNW FET biosensors discussed in this section were selected to exemplify a range of design approaches and their applications in wearable biosensing. These sensors were chosen for their advancements in sensitivity, specificity, and integration, addressing critical challenges in wearable diagnostics. The SiNW FET for tuberculosis detection was included for its CMOS compatibility and high sensitivity, while the exosome detection biosensor highlights integration with microfluidic systems. The kidney failure biomarker sensor was featured to demonstrate the potential for achieving exceptional sensitivity and specificity with SiNW FETs. Thus, SiNW FETs with diverse configurations are ideally suited for the highly specific and sensitive detection of biomolecules.

#### 2.1.2. Si-Based ISFET Biosensors

**Figure 3:**
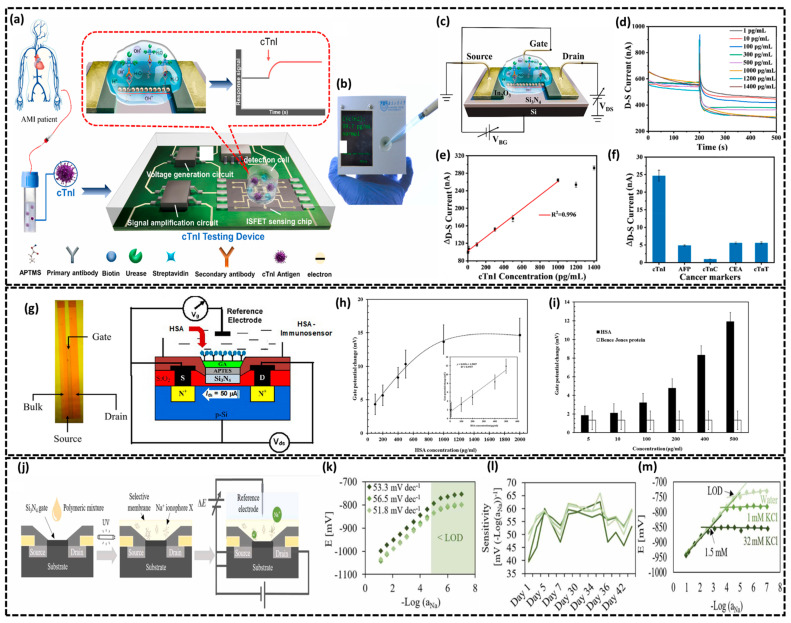
(**a**) Schematic representation of the detection mechanism implemented in the ISFET-based handheld testing device for cTnI measurement. (**b**) Photograph of the ISFET-based handheld testing device utilized for the detection of cTnI. (**c**) Electrode connection configuration of ISFET in the dual-gate mode. (**d**) IDS current response to varying cTnI concentrations. (**e**) Linear curve of varying cTnI concentrations ranging from 1 pg/mL to 1000 pg/mL. (**f**) Specificity test of ISFET biosensor with four different biomarkers. (**a**–**f**) reproduced with permission from Elsevier, Copyright [2021]. (**g**) Top view and cross-sectional view of Si_3_N_4_-ISFET biosensor. (**h**) Relationship between anti-HSA antibody at various concentrations and gate potential change in ISFET. (**i**) Specificity of has-modified ISFET biosensor for the detection of the HSA protein. (**g**–**i**) reproduced from [71] under Creative Commons Attribution License, 2021. (**j**) Cross-sectional schematic of the Na^+^ ISFET illustrating membrane deposition procedure and the biosensing mechanism. (**k**) Calibration plots of Na^+^ ISFETs over the varying concentration range. (**l**) The sensitivity of four ISFETs periodically calibrated over a duration of 43 days. (**m**) Calibration plots of a Na^+^ ISFET obtained in deionized water and in KCl solutions with varying concentrations. (**j**–**m**) reproduced with permission from Elsevier, Copyright [2023].

Silicon-based ion-sensitive field-effect transistors (ISFETs) are a versatile class of biosensors leveraging CMOS technology and the unique properties of silicon for highly sensitive and miniaturized biochemical sensing [43,68]. ISFETs derived from modifications to conventional MOSFETs which operate by sensing changes in ionic concentrations or surface potentials thus allowing for real-time, label-free detection of analytes like pH, DNA, proteins, and different biomarkers [69]. Their integration with silicon microfabrication has resulted in compact, cost-effective, and scalable platforms which makes them ideal for point-of-care diagnostics. This section explores their design, operation, and recent advancements in biosensing applications. In this regard, Wang et al. fabricated a highly sensitive ISFET biosensor on a silicon substrate for the rapid detection of cardiac troponin I (cTnI) which paved the way for diagnosing acute myocardial infarction (AMI), a life-threatening cardiovascular condition as depicted in schematic overview of bio-ISFET in Figure 3a,b [70]. The bio-ISFET based on an In_2_O_3_ nanobelt channel was developed with a dual-gate structure through CMOS-integrated microfabrication techniques. The back-gate voltage (V_BG_) was applied via the silicon substrate with the built-in reference electrode connected to the source and a forward bias voltage applied between the source and drain as shown in Figure 3c. The sensor surface was carefully functionalized to enable selective cTnI detection. Following ultrasonic cleaning, 2 wt% APTMS was applied to introduce amino groups on the surface via covalent bonding with the In_2_O_3_, followed by air drying for 12 h. A mixture of 0.3 M EDC and 0.1 M NHS activated the carboxyl groups of cTnI primary antibodies (1 mg/mL) for covalent attachment, forming stable amide bonds. After incubating at room temperature for 1 h and storing at 4 °C for 12 h, the surface was blocked with 1% BSA to minimize nonspecific binding, followed by PBS rinsing. After washing, urea was introduced for electrochemical cTnI detection which was diffusion-controlled and dependent on target concentration as depicted in Figure 3d, with the drain–source current gradually increasing as cTnI concentrations rose from 1 pg/mL to 1000 pg/mL. Figure 3e illustrates a linear relationship between Δ_drain-source current_ and cTnI concentration (1–1000 pg/mL) with high accuracy (R^2^ = 0.9996), demonstrating excellent sensitivity of 132 pA mL·ng^−1^ and an LOD of 0.3 pg/mL while concentrations above 1000 pg/mL exhibited non-linearity. Lastly, the authors tested the device’s selectivity for cTnI against four nonspecific biomarkers (AFP, CEA, cTnC, and cTnT), showing minimal current changes (5.03–5.65 nA) compared to cTnI’s response (24.80 nA) demonstrating its high specificity (Figure 3f). Later, Saengdee et al. developed a label-free Si_3_N_4_-ISFET-based immunosensor to detect human serum albumin (HSA) in human urine as an early indicator of kidney damage [71]. Figure 3g shows the top-view and cross-sectional structure of the Si_3_N_4_-ISFET consisting of n-doped source and drain regions in a p-type substrate with dimensions of 2000 × 100 μm. A 30 nm Si_3_N_4_ layer is grown between the source and drain, and an Ag/AgCl reference electrode was integrated. The Si_3_N_4_-ISFET surface was functionalized with APTES and GA for anti-HSA antibody immobilization (Figure 3g). The dose–response curve (Figure 3h) shows a gate potential change proportional to HSA concentrations from 5 to 500 μg/mL, with an LOD of 5 μg/mL and a correlation coefficient of R^2^ = 0.9757, while saturation occurs above 1000 μg/mL due to the binding limitation of immobilized anti-HSA antibodies. The specificity of the HSA immunosensor was tested by comparing the gate potential changes for HSA and Bence Jones proteins at concentrations from 0 to 500 μg/mL as illustrated in Figure 3h. The gate potential for the HSA protein was significantly higher than that for Bence Jones protein indicating good selectivity of the immunosensor. In another study, Rovira et al. developed a sodium (Na^+^) ISFET biosensor for sweat analysis by fabricating biochips on SOI (silicon-on-insulator) wafers which serve as the transistor channel and electrically isolate the ISFET from the substrate and the Si_3_N_4_ gate [72]. As displayed in biosensor’s schematic in Figure 3j, a Na^+^-selective membrane mixture was prepared using photocurable polymers drop-casted onto the ISFET gate and patterned to the required region using photolithography. An Ag/AgCl reference gate electrode was incorporated to ensure stable and accurate potential measurements as shown in Figure 3j. The Na^+^ ISFETs were tested across NaCl concentrations (10^−7^ M to 1 M) without interfering ions showing an LOD of 9.4 × 10^−6^ ± 4.9 × 10^−6^ M and a Nernstian slope of 53.9 ± 2.4 mV (−Log aNa)^−1^ within a linear range of 3.2 × 10^−5^ M to 1 M NaCl (Figure 3k). The sweat biosensor demonstrated a reproducible sensitivity of 60.7 ± 0.5 mV (−Log aNa)^−1^ exhibiting high repeatability and stable performance with a lifetime of up to one month (Figure 3l). Finally, the selectivity of the Na^+^ ISFET biosensor was tested against potassium (K^+^), known as a potential interferent in sweat. Figure 3i shows calibration curves in deionized water and KCl solutions, with a selectivity coefficient (Kpot Na, K) of 0.0695. While K^+^ interference increases above 1 mM, Na^+^ detection remains unaffected above 1.5 mM due to the higher Na^+^ concentration in sweat hence confirming the biosensor’s high selectivity towards Na^+^ ions. From this discussion various applications of Si-based ISFET biosensors have been reviewed including applications in cardiac marker detection, renal biomarker monitoring, and electrolyte analysis focusing their exceptional sensitivity, specificity, and adaptability on diverse analytes. With ongoing advancements in functionalization strategies and device architectures, ISFET biosensors hold immense promise for next-generation diagnostics.

#### 2.1.3. Si-Based Hydrogel-Gated FET Biosensors

**Figure 4:**
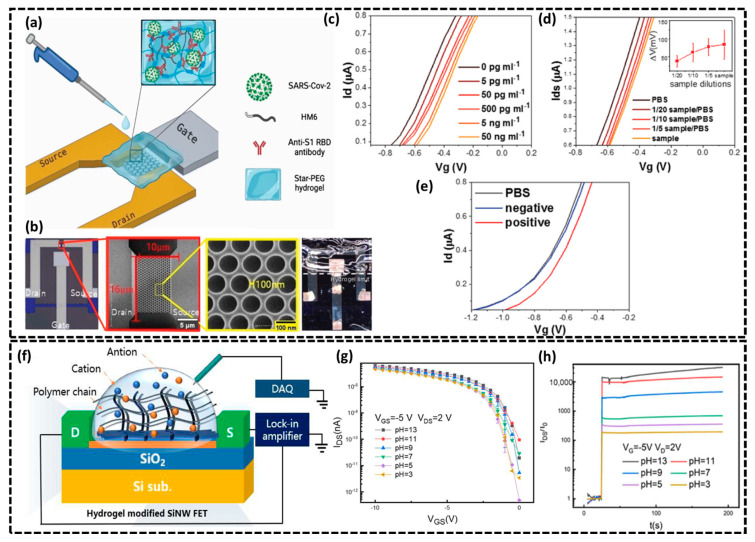
(**a**) Schematic illustration of hydrogel-gated FET SARS-CoV-2 biosensor. (**b**) (Left to right) Optical microscopy of unmodified FETs, SEM magnification of the nanonet, and hydrogel-modified FET. (**c**) Biosensor response to SARS-CoV-2 RBD in PBS. (**d**) Response to RBD in saliva, with the calibration graph included as an inset. (**e**) Transfer characteristics of the biosensor after incubation with samples from COVID-19 negative (blue) and COVID-19 positive (red) cases. (**a**–**e**) reproduced from [75] under Creative Commons Attribution License, 2023. (**f**) Schematic showing biosensing mechanism of hydrogel-modified SiNW FET. (**g**) Transfer characteristic curves of hydrogel-functionalized SiNW FET at varying pH concentrations. (**h**) Real-time response of IDS for a hydrogel-functionalized SiNW FET measured in solutions of varying pH levels. (**f**–**h**) reproduced from [76] under Creative Commons Attribution License, 2022.

Silicon-based hydrogel-gated FET biosensors are gaining significant attention due to their ability to detect biomolecules with high sensitivity and continuous tracking [73,74]. These sensors integrate the properties of silicon with hydrogels which respond to environmental changes hence leading to precise monitoring of biological interactions. This section explores the fundamental principles, recent progress, and practical uses of silicon-based hydrogel-gated FET biosensors in modern biosensing technology. In this regard, Parichenko et al. fabricated a hydrogel-gated silicon nano-FET transistor for the quantitative detection of coronavirus 2 (SARS-CoV-2) by employing star-shaped polyethylene glycol (PEG)-based hydrogel as host bioreceptor as depicted in the schematic of the biosensor (Figure 4a) [75]. The authors utilized the FET structure for nanobiosensor fabrication having a silicon nanonet as a channel with a 100 nm lateral distance at the interconnects (see SEM in Figure 4b). Biofunctionalization was achieved through in situ polymerization of the star-PEG-heparin hydrogel loaded with antibodies against the SARS-CoV-2 spike protein. Heparin maleimide (HM6) and PEG-thiol were combined with antibodies (1.6 μg/mL), and the resulting solution was applied to the FET surface. Polymerization proceeded for 60 min in a humidified atmosphere under a glass slide to ensure a flat, homogeneous surface. The hydrogel was then hydrated with PBS, creating a stable 3D matrix that efficiently immobilized antibodies and enhanced biosensor sensitivity in physiological conditions. The biosensing measurements were conducted in PBS solutions with RBD concentrations ranging from 5 pg/mL to 50 ng/mL, as shown in Figure 4c. The PEG hydrogel-gated FET exhibited a sensitivity of 30 mV ± 5.7 mV to ten times increase in RBD concentration, while the biosensor in diluted buffer showed a sensitivity of 31 mV ± 3.5 mV. These results demonstrate that the hydrogel layer maintains sensitivity in high ionic strength solutions. The author further evaluated the biosensor’s performance by exposing it to real SARS-CoV-2 viral samples. Due to inconsistent results with β-propiolactone (BPL) deactivation, the samples were heated at 80 °C for 1 h, which produced consistent I-V curves with concentration-dependent shifts (Figure 4d). Moreover, a 105 mV shift was observed with the COVID-19 positive sample, similar to ≈1 ng/mL of SARS-CoV-2 RBD while no shift was recorded for the negative sample (Figure 4e), which validates the biosensor’s ability to detect virus presence in clinical samples. In another study, Li et al. reported a pH-sensitive hydrogel-functionalized SiNW FET transistor by utilizing an acrylamide hydrogel layer that responds to changes in pH leading to the change in conductivity of the SiNW [76]. Figure 4f illustrates the schematic of a hydrogel-modified SiNW FET with HfO_2_/SiO_2_ layers employing SiNW arrays between source and drain electrodes where each SiNW measuring 25.86 nm in width and 36.39 nm in height. The gate dielectric layer fully encapsulates the silicon wire hence preventing leakage current and maintaining a stable liquid gate environment. To evaluate the pH sensing performance, the gate-sensitive region of the hydrogel-coated SiNW FET was briefly immersed in a buffered pH solution. As shown in transfer characteristics curves (Figure 4g), the biosensor exhibited drain current changes with increasing pH, resulting in a shift in the threshold voltage and exhibited high pH sensitivity of 100 mV/pH. The pH sensing can be calculated as follows:

(3) $$S=\frac{V_{th1}-V_{th2}}{pH_{1}-pH_{2}}$$

where *S* represents the sensor’s sensitivity, *V*_*th*1_ corresponds to the threshold voltage at lower pH (*pH*_1_), and *V*_*th*2_ represents the threshold voltage at higher pH (*pH*_2_). As the pH increases from 3 to 13, the drain current exhibits a corresponding increase, resulting in a shift in the threshold voltages. The threshold voltage values for the hydrogel-modified SiNW FET were determined using the constant current methods, as reported in previous studies [77,78]. The authors also tested the real-time pH sensing performance of hydrogel-functionalized SiNW FET with drain current sharply increases when sensor gate is exposed to varying pH liquid concentrations as illustrated in Figure 4h. In summary, silicon-based hydrogel-gated FET biosensors represent a significant and reliable approach for achieving highly sensitive and selective biosensing solutions. By integrating hydrogels with FETs, these biosensors can effectively detect various biomolecules through electrochemical interactions with the hydrogel layer enhancing biocompatibility and enabling high pH-responsive sensing.

#### 2.1.4. Si-Based TFET Biosensor

**Figure 5:**
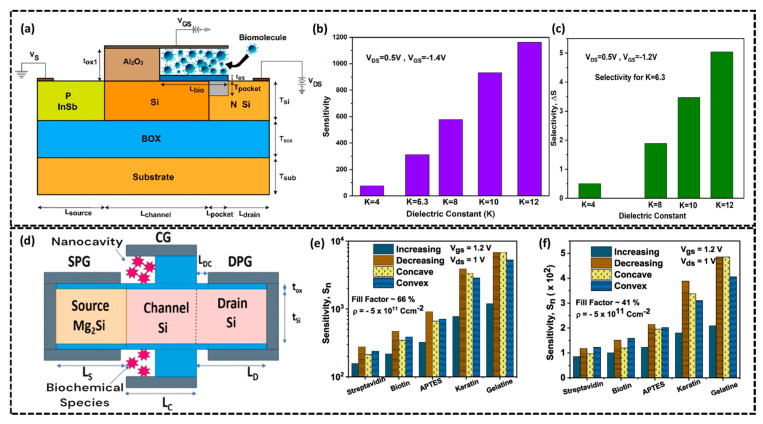
(**a**) Schematic diagram of InSb/Si TFET-based biomolecule sensor. (**b**) Impact of dielectric constant on sensor sensitivity. (**c**) Selectivity versus dielectric constant. (**a**–**c**) reproduced with permission from Elsevier, Copyright [2022]. (**d**) Cross-sectional view DM-DL-DGTFET biosensor. (**e**) Sensitivity of various step profiles for partly filled cavity at a 66% fill factor, and (**f**) ~41% fill factor for DL-DGTFET biosensor. (**e**,**f**) reproduced with permission from Elsevier, Copyright [2024].

Silicon-based tunnel field-effect transistor (TFET) biosensors have emerged as a promising technology for high-sensitivity biosensing applications [79]. These devices operate based on the quantum tunneling effect, a quantum-mechanical phenomenon in which charge carriers traverse a potential energy barrier without requiring thermal excitation. This effect arises due to the wave–particle duality of electrons, enabling carrier transport through narrow energy barriers in nanoscale devices. In TFETs, band-to-band tunneling at the source–channel junction facilitates ultra-low voltage operation, thereby enhancing sensitivity in biomolecular detection [80]. Furthermore, TFET biosensors utilize advanced silicon fabrication methodologies, offering a highly scalable and cost-efficient platform. This has stimulated significant research efforts towards their integration in various bio-detection systems. In this regard, Swain et al. designed a TFET biosensor utilizing an InSb/Si heterojunction structure for biomolecule detection [81]. The schematic in Figure 5a depicts the proposed InSb/Si TFET biosensor, comprising an InSb source, a silicon channel, and a silicon drain with a nanocavity positioned near the gate region. The biofunctionalization of the InSb/Si TFET biosensor was accomplished by constructing a nanocavity with an adhesive SiO_2_ layer to promote biomolecule attachment. A high-k dielectric Al_2_O_3_ layer was deposited using atomic layer deposition at 300 °C to improve sensitivity and provide surface passivation. The Al_2_O_3_ layer was selectively etched to uncover the SiO_2_ adhesive surface, creating a functionalized nanocavity for biomolecule immobilization, facilitating label-free biosensing through dielectric modulation. The dielectric properties of biomolecules within the nanocavity influence the device’s ambipolar current. This TFET biosensor configuration uses Al_2_O_3_ as a high-k gate dielectric to enhance electrical performance and sensitivity by modulating the ambipolar current based on the dielectric constant of the biomolecules introduced into the cavity. Figure 5b illustrates the effect of the dielectric constant on sensitivity, with the device achieving a maximum sensitivity of 1193 at a dielectric constant K = 12 and a gate-source voltage (V_GS_) of −1.4 V. The biosensor’s selectivity, depicted in Figure 5c, increases with higher dielectric constants and reaches a maximum of 5.07 at K = 12, demonstrating its ability to differentiate biomolecules based on their dielectric properties. Moreover, Goyal et al. proposed a Mg_2_Si/Si heterojunction-based dopingless dielectric-modulated double-gate TFET (DM-DGTFET) biosensor for detecting neutral and charged biomolecules [57]. Figure 5d shows the biosensor schematic, and consists of a staggered type-II heterojunction formed at the Mg_2_Si source and silicon channel interface. This design enables efficient tunneling by minimizing the barrier width and improving ON current. The biosensor includes a single nanocavity (20 nm × 5 nm) etched beneath the gate oxide near the source–channel junction to capture biomolecules. A 1 nm thick SiO_2_ immobilization layer is used to confine analytes within the cavity which ensures accurate detection. The cavity’s proximity to the tunneling junction enhances capacitive coupling, significantly impacting the device’s electrical characteristics. The drain current sensitivity of the biosensor was determined by sequentially filling the cavity with neutral biomolecules of varying dielectric constants (k = 2.1, 2.63, 3.57, 8, 12) and negatively charged biomolecules (e.g., DNA) with dielectric constant (k = 8) and charge density (ρ = −1 × 10^−12^ cm^−2^. The sensitivity (*S_n_*), is obtained as follows:

(4) $$S_{n}=\frac{I_{ON,k}}{I_{ON,air}}{|}_{V_{gs}=0.55V,V_{ds}=1V}$$

where *I_ON_*, *k*, and *I_ON_*, air represent the ON-state currents with and without biomolecules in the cavity, respectively. Finally, authors investigated the biosensor response towards various biomolecules considering the practical scenario of non-uniform biomolecule distribution due to steric hindrance. Figure 5e,f analyze the sensitivity of the biosensor for different step profiles (increasing, decreasing, concave, convex) at fill factors of 66% and 41%, respectively. Maximum sensitivity is achieved with decreasing or concave profiles which maximize biomolecule presence near the source–channel junction, enhancing gate-to-channel coupling and capacitance. For lower dielectric constants (e.g., APTES), convex profiles perform better, while concave profiles dominate at higher dielectric constants. Sensitivity at a 66% fill factor is 13.08 times greater than at 41%, driven by enhanced electron tunneling at the source–channel interface. In conclusion, Si-based TFET biosensors offer unique combination of high sensitivity, low power consumption, and miniaturization makes them excellent candidates for the development of advanced and reliable biosensing devices. With further advancements in device design and material engineering, Si-based TFET biosensors can play a crucial role in enhancing the precision and efficiency of biomolecule sensing systems.

#### 2.1.5. Si-Based Capacitive Biosensors

**Figure 6:**
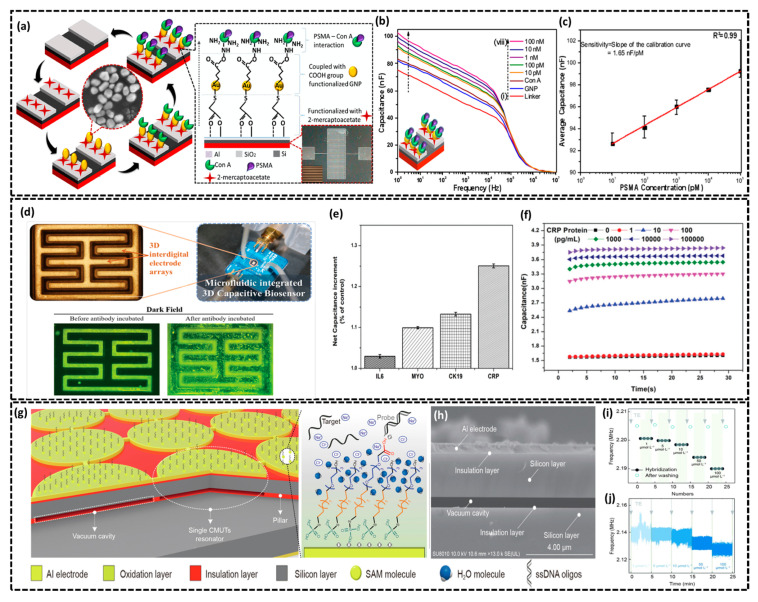
(**a**) Stepwise fabrication process and schematic illustration of the lectin biosensor: (anti-clockwise) bare Al-IDE; surface modification with linker 2-mercaptoacetate, forming a SAM layer with thiol and carboxylate functional groups; deposition of carboxylic acid-functionalized GNP; immobilization of Con A; detection of the target PSMA. (**b**) Capacitance as a function of logarithmic frequency for Al-IDE, modified with (i) linker, (ii) GNP, (iii) Con A, and (iv–viii) various concentrations of PSMA; (**c**) calibration curve utilizing capacitance data for varying concentrations; (**a-c**) reproduced with permission from Elsevier, Copyright [2021]. (**d**) Micrograph of 3D interdigital electrode arrays (top left), image of the microfluidic-integrated 3D capacitive biosensor (top right), and fluorescent analysis (before and after) of biofunctionalization on gold electrodes following biological modification and antibody incubation. (**e**) Capacitance responses to reference biomolecules and CRP. (**f**) Real-time monitoring measures the variation in capacitance value of the electric sensor as the CRP concentration increases. (**d**–**f**) reproduced with permission from Royal Society of Chemistry, Copyright [2021]. (**g**) Schematic of a CMUTs-based label-free biosensor with Al electrodes designed to detect ssDNA oligonucleotides utilizing a self-assembled monolayer (SAM). (**h**) Cross-sectional SEM micrograph of fabricated CMUTs cell. (**i**) Frequency responses with various concentrations of complementary ssDNA measured by the impedance analyzer, and (**j**) internal ASIC interface. (**g**–**j**) reproduced from [85] under Creative Commons Attribution License, 2024.

Silicon-based capacitive biosensors are another class of silicon biosensors, known for their high sensitivity, scalability, and compatibility with modern electronics. These biosensors utilize silicon’s excellent conductivity, adjustable surface properties, and advanced fabrication methods to combine functional materials such as self-assembled monolayers, nanoparticles, and bioreceptors to precisely detect targets like proteins, DNA, and glycoproteins, including the prostate-specific membrane antigen (PSMA) [82]. Subramani et al. presented a silicon-based capacitive biosensor for the detection of the PSMA, a promising biomarker for prostate cancer diagnosis [83]. As detailed in the stepwise fabrication schematic of the biosensor (Figure 6a), the sensor employs aluminum-interdigitated electrodes (Al-IDEs) on a p-type silicon wafer (resistivity of 100 Ω cm), modified with a self-assembled monolayer (SAM) of 2-mercaptoacetate, functionalized gold nanoparticles (GNPs) and Concanavalin A (Con A) as the bioreceptor. The biofunctionalization process (see Figure 6a) begins with the formation of the SAM on the Al-IDEs through 2-mercaptoacetate, providing carboxylate and thiol functional groups as the foundation for subsequent biomolecule immobilization. Carboxylic-functionalized GNPs are then deposited via Au-S bonding to enhance conductivity and support lectin conjugation. Con A is immobilized onto the GNP-modified surface using EDC/NHS chemistry, where it acts as a bioreceptor for mannose residues in PSMA, ensuring high specificity and accuracy in biomolecule detection. Finally, ethanolamine is used as a blocking agent to prevent nonspecific binding, completing the surface modification process. Figure 6b presents the capacitance–frequency graph for Al-IDEs channels modified with a linker COOH-GNP and Con-A, showing incremental capacitance values of 75.3 nF, 81.9 nF, and 83.3 nF at 1 Hz (Figure 6bi–iii). Further increases in capacitance are observed as the Con A-immobilized biosensor detects varying concentrations of PSMA with the lectin–glycoprotein interaction increasing molecular size which alters charge distribution and enhances dielectric properties, hence leading to continuous capacitance growth. The calibration plot depicting the relationship between the change in capacitance (ΔCapacitance) and PSMA concentration (Figure 6c) demonstrates the biosensor’s capability for the linear detection of PSMA in the range of 10 pM to 100 nM. The biosensor exhibits a high sensitivity of 2.64 nF/M, a correlation coefficient of 0.98, and a detection limit of 10 pM. Later, Liu et al. proposes a microfluidic-integrated 3D capacitive biosensor for real-time and label-free detection of the C-reactive protein (CRP) biomarker [84]. As illustrated in Figure 6d, the 3D capacitive biosensor was fabricated using the open cavity molding method by integrating 3D interdigital gold electrodes on a PCB with a microfluidic channel. SU8 photoresist was patterned on a silicon wafer via photolithography followed by bonding of molded PDMS to the electrodes using plasma treatment to create inlet and outlet ports. The 3D capacitive biosensor was biofunctionalized with anti-CRP antibodies immobilized using mixed sulfhydryl molecules (11-mercaptoundecanoic acid and 6-mercapto-1-hexanol) and EDC/sulfo-NHS activation. Successful biofunctionalization was confirmed through fluorescent characterization with bright and clustered fluorescent spots observed under the dark field (see Figure 6d). Further, the specificity of the anti-CRP-functionalized 3D capacitive biosensor was confirmed by testing non-target proteins (CK19, MYO, IL-6, and PBS) alongside CRP by keeping each at the same concentration of 100 ng/mL. CRP exhibited a significantly higher capacitance response compared to non-target proteins, demonstrating high specificity due to the antibody–antigen binding that increased the dielectric constant, as shown in the bar graph of Figure 6e. The sensitivity of the capacitive biosensor was evaluated by sequentially injecting CRP antigen solutions at six concentrations (1–100,000 pg/mL) into the microfluidic channel with the LOD recorded as 1 pg mL^−1^. Real-time capacitance measurements were monitored using an LCR meter at 200 mV and frequencies from 20 Hz to 1.5 MHz showed a positive correlation between capacitance values and CRP concentration, as depicted in Figure 6f. In another recent reported study, Li et al. reported a capacitive micromachined ultrasonic transducers (CMUTs)-based resonant biosensor by employing advanced MEMS and CMOS techniques to achieve high sensitivity and miniaturization for detecting single-stranded DNA (ssDNA) [85]. The biosensor operates in resonant mode where a DC bias and AC voltage generate electrostatic forces that induce vibrations in the membrane, hence enabling precise detection. As shown in Figure 6g, the CMUT arrays were fabricated using 350 °C direct bonding with a 450 nm cavity height followed by a 100 nm SiO_2_ insulating layer to prevent short circuits and a 2 µm SOI-based silicon membrane (see SEM image in Figure 6h), which ensures structural integrity and enhances the device’s electrical and mechanical performance. The resonant frequency *f_mem_* of a single CMUT cell was calculated as follows:

(5) $$f_{mem}=\frac{0.83t_{mem}}{R_{mem}^{2}}\sqrt{\frac{E}{\pi \rho (1-\nu^{2})}}$$

where *R_mem_* and *t_mem_* represent the radius and thickness of the top membrane, respectively; *E* denotes the Young’s modulus, *v* is the Poisson ratio, and *ρ* refers to the density. For silicon, *E* = 169 GPa, *v* = 0.28, and *ρ* = 2332 kg/m^3^q. Furthermore, the biosensor was functionalized with a self-assembled monolayer of ethylene-glycol thiol-phosphate acid on the CMUTs’ Al electrodes improving water molecule adjustment and enhancing ssDNA probe selectivity (see Figure 6g). A notable area ratio difference between complementary DNA (40.40%) and non-complementary DNA (0.10%) at 100 µmol∙L^−1^ was achieved. The authors implemented an internal application-specific integrated circuit (ASIC) interface oscillator and external impedance analyzer to measure the sensitivity and LOD of varying complementary ssDNA concentrations as illustrated in Figure 6i,j. The capacitive biosensor demonstrated a mass detection sensitivity of 0.061 fg∙Hz^−1^∙µm^2^ and an LOD of 0.635 µmol∙L^−1^, showing significantly enhanced sensitivity compared to conventional methods. In conclusion, the fabrication of highly sensitive and selective diagnostic tools may be achieved through the use of silicon-based capacitive biosensors. Their scalability, flexibility to various surface functionalization processes, and exceptional electrical qualities make them perfect for precise biomolecule detection.

### 2.2. Si-Based Optical and Photonic Biosensors

Another category of silicon biosensors are optical and photonic biosensors that have been widely studied for their high sensitivity and robust biomolecule detection capabilities [86]. These biosensors exploit the unique optical properties of silicon such as its high refractive index and compatibility with existing microfabrication techniques to enable precise monitoring of molecular interactions [87]. Operating on the principle of light–biomolecule interaction, these biosensors utilize infrared or visible light transmitted through silicon waveguides. Biomolecule binding alters the local refractive index, affecting light propagation. This change is detected through variations in intensity, phase, or wavelength, enabling quantitative biomolecular analysis. Key configurations include micro-ring resonators, photonic crystals, and interferometric sensors. Micro-ring resonators exploit resonance shifts upon biomolecule binding, correlating with molecular concentration, whereas photonic crystal sensors leverage periodic structures to induce spectral shifts upon environmental changes, facilitating biomolecule quantification. Interferometric sensors detect phase differences due to refractive index changes, enabling highly sensitive biomolecular interaction monitoring. In all of these configurations, changes in optical properties such as shifts in resonance wavelength, phase, or intensity are continuously monitored. These optical signals are then processed to quantify the amount of target biomolecule in the sample, allowing for highly sensitive and accurate detection of biomolecules like proteins, DNA, or small metabolites. This section explores the fundamentals, recent developments and practical uses of silicon-based optical and photonic biosensors while highlighting their significance in the latest biosensing technologies.

**Figure 7:**
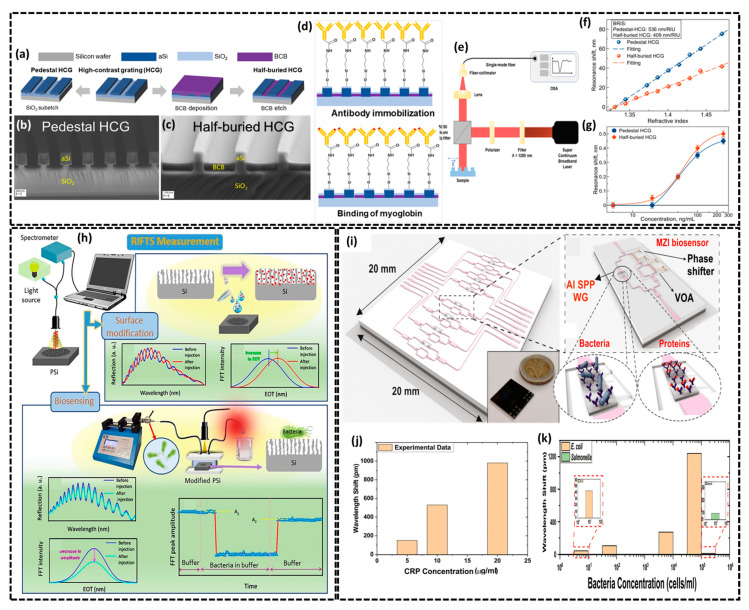
(**a**) Schematic illustrating the fabrication steps for the half-buried and pedestal HCG structures. Cross-sectional SEM images of (**b**) the pedestal HCG and (**c**) the half-buried HCG are shown. (**d**) Immobilization of the myoglobin antibody and myoglobin binding to the biosensor surface. (**e**) Optical setup: the optical spectrum analyzer (OSA) acts as the detector, while a broadband supercontinuum laser serves as the light source. (**f**) Resonance wavelength of the pedestal (blue) and half-buried (red) HCGs as a function of refractive index (RI) changes caused by varying glycerol concentrations. (**g**) Resonance shifts measured between the myoglobin antibody (blank) and myoglobin molecules (antigen). (**a**–**g**) reproduced with permission from the American Chemical Society, Copyright [2023]. (**h**) Schematic of RIFTS measurement: the surface modification section includes analyte molecules that penetrate or alter surface chemistry, changing EOT, reflection spectra from PSi at each step, and FFT intensity from the spectra. The biosensing section includes bacterial suspension monitored with a fluidic system, reflection spectra during two steps, FFT intensity from the spectra, and a real-time biosensor response during washing, bacterial exposure, and final washing, reproduced from [89] under Creative Commons Attribution License, 2020. (**i**) (left) Schematic of the photonic biochip (inset shows a photo of the biochip with a two Euro coin for scale comparison), (right) schematic layout of MZI biosensors, with insets showing the capture of pathogens and proteins on the Al) surface. (**j**) Absolute wavelength shift for each CRP concentration. (**k**) Bar graph of the wavelength shift for varying bacteria concentration. (**i**–**k**) reproduced with permission from Elsevier, Copyright [2024].

In this context, Leonid Yu et al. reported a Si-based optical biosensors for the detection of myoglobin (biomarker for diagnosing acute myocardial infarction) featuring two different high-contrast grating (HCG) configurations, pedestal and half-buried HCGs, fabricated at the nano-level scale [88]. These varying HCG nanostructures displayed improved efficiency over traditional HCGs offering greater volume and surface sensitivity for the better detection of analytes. Figure 7a–c illustrates the fabrication process and SEM images of pedestal and half-buried HCGs optical biosensors. The authors carried out the biofunctionalization process (see Figure 7d) through a series of precisely optimized surface modifications to enable highly specific and efficient myoglobin detection. First, a 5 nm SiO_2_ layer was deposited on the HCG structures to protect the underlying benzocyclobutene polymer and provide a suitable surface for functionalization. The surface was then hydroxylated for 60 min using piranha solution (a 4:1 mixture of sulfuric acid and hydrogen peroxide) to introduce hydroxyl groups. Silanization was performed with aminopropyltrimethoxysilane (APTMS), resulting in amino-functionalized surfaces. Subsequently, myoglobin antibodies were immobilized on the surface using EDC/NHS chemistry by incubating the samples in a solution containing 200 mM EDC and 50 mM NHS for 60 min. Finally, myoglobin was introduced at various concentrations (5 to 250 ng/mL) and allowed to bind to the immobilized antibody for 30 min, enabling specific detection through resonance shift measurements. To perform optical biosensing, free-space reflectance measurements were performed using a broadband supercontinuum laser as the light source and an optical spectrum analyzer as the detector with a resolution of 0.1 nm and results averaged over 10 scans (Figure 7e) while bulk refractive index sensitivity (BRIS) measurements were carried out to assess the biosensing capabilities of the pedestal and half-buried HCGs biosensors. BRIS is given by the following:

(6) $$S_{B}=\frac{\Delta \lambda }{\Delta n}[nm/RIU]$$

Here, Δλ represents the shift in resonance wavelengths due to a change in the bulk refractive index where the refractive index change is denoted as Δ*n*. BRIS was assessed using glycerol–water solutions (RI 1.33–1.47 RIU) showing linear resonance shifts with sensitivities of 536 nm/RIU for pedestal HCGs and 409 nm/RIU for half-buried HCGs. The higher sensitivity half-buried HCGs is due to their greater surface exposure (Figure 7f). For myoglobin detection, reflectance spectrum shifts were measured using functionalized HCG structures with myoglobin concentrations of 5–200 ng/mL. Detection curves in Figure 7g for pedestal and half-buried HCGs showed sigmoidal responses, with limit of detection values of 38.5 ng/mL and 35.7 ng/mL and limit of quantification values of 143 ng/mL and 105 ng/mL, respectively. Half-buried HCGs demonstrated superior surface sensitivity and a lower LOD for myoglobin biosensing. Also, Mona et al. investigated the unique optical biosensing properties of porous silicon (PSi) by fabricating a lectin-functionalized PSi-based biosensor for real-time and label-free detection of *E. coli* and *S. aureus* bacteria employing reflectometric interference Fourier transform spectroscopy (RIFTS) measurement system [89]. The surface modification of PSi with lectin bioreceptors (ConA, WGA) and the bacteria detection mechanism via the RIFTS system are detailed in Figure 7h. RIFTS measurements utilized a bifurcated optical fiber system to direct light from a tungsten-halogen source onto the sample surface with the reflected beam analyzed by a spectrometer (see Figure 7h). Reflectance spectra were recorded within the range of 400–1000 nm and processed using fast Fourier transformation (FFT) to obtain effective optical thickness values (2 × the product of the refractive index and the thickness measurement of the porous film, Figure 7h (surface modification section)). Surface parameters such as porosity, the refractive index, and thickness were calculated using the spectroscopic liquid infiltration method, as depicted in Figure 7h. For biosensing, modified PSi samples were mounted on a fluidic system (see biosensing section of Figure 7h), enabling saline buffer and bacterial solutions to flow over the surface. Bacterial suspensions (10^−3^ to 10^−5^ cells/mL) were incubated for 25–30 min at ambient temperature with reflective spectra recorded every 2 min. FFT peak amplitude changes before and after bacterial exposure quantified biosensing performance, as illustrated in Figure 7h (biosensing section). FFT peak can calculated as follows:

(7) $$FFT\;peak\;amplitude\;change(\%)=\frac{A_{1}-A_{2}}{A_{1}}\times 100$$

Here, *A*_1_ and *A*_2_ represent the average *FFT* peak amplitudes before and after bacterial exposure, respectively, highlighting the changes in the reflectance spectrum due to bacterial binding. The biosensor’s response differed for the two bacterial types based on the lectin used. PSi modified with ConA showed the strongest response to *E. coli*, while WGA modification was the most effective for detecting *S. aureus* under the same conditions. ConA shows the best sensitivity to *E. coli* and WGA to *S. aureus* with a linear response from 3 × 10^3^ to 3 × 10^5^ cells/mL and a low LOD of 10^3^ cells mL^−1^ was observed in both cases. Furthermore, Manolis et al. presented a multifunctional diagnostic silicon photonic biochip for the *E. coli* (a commonly encountered pathogen) and CRP (a C-reactive protein, produced by the liver in response to inflammation) [90]. Figure 7i depicts the photonic biochip compatible with CMOS fabrication and supports multiple biosensors for multiple detections. Each biosensor features a 70 μm aluminum (Al) plasmonic stripe that acts as a sensing transducer, detecting changes in the refractive index of the surrounding medium and integrated within a Si_3_N_4_ waveguide-based Mach–Zehnder interferometer (Figure 7i). The aluminum sensing surfaces of biosensor were functionalized with Anti-CRP and Anti-*E. coli* as biorecognition probes. The photonic biosensor’s detection capability was evaluated by reacting increasing CRP concentrations (0 to 20 μg/mL) with the Al channel which results in larger observed wavelength shifts as CRP concentration increased as shown in Figure 7j. The sensitivity and LOD were calculated as 50.8 ± 4.5 pm/μg/mL and 0.29 μg/mL, respectively. For *E. coli* detection, the immobilization of anti-*E. coli* antibodies on the Al channel enabled the photonic biosensor to detect bacterial concentrations ranging from 10 to 1000 cells/mL. *E. coli* samples were flowed through the sensor channel and wavelength shifts were observed with increasing concentrations (see Figure 7k). The detection limit was determined to be 10 cells/mL and the biosensor’s sensitivity was demonstrated by its ability to detect a single bacterium with an observed wavelength shift (42 pm) closely matching the predicted value (50 pm). In summary, silicon-based optical and photonic biosensors take advantage of silicon’s inherent features to achieve high sensitivity and specificity in biomolecule detection. Significant inventions such as high-contrast gratings, porous silicon, and micro-ring resonators have improved their applications in diagnostics, pathogen detection, and cancer biomarker analysis, suggesting considerable potential for future healthcare technology.

### 2.3. Si-Based Microcantilever Biosensors

**Figure 8:**
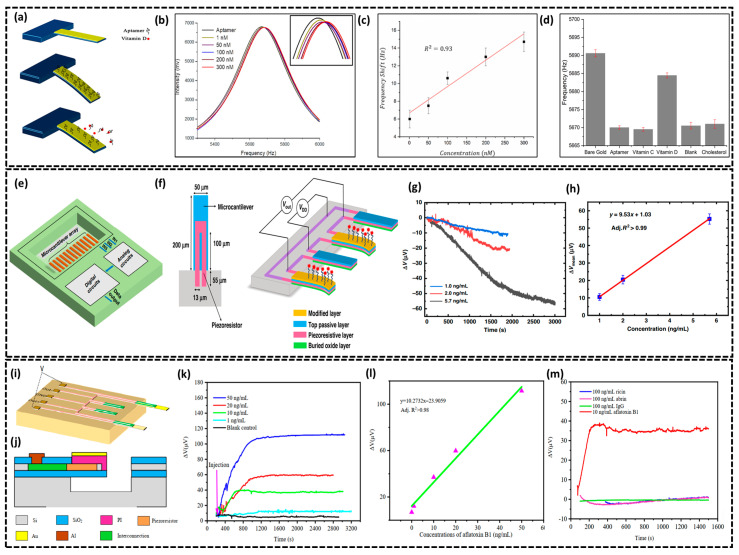
(**a**) Schematic showing the biosensing principle of the microcantilever biosensor. (**b**) Time-dependent resonance frequency curves of aptamer-functionalized microcantilevers exposed to varying concentrations of vitamin D. (**c**) Calibration plot showing the frequency shift in the aptamer-functionalized microcantilever relative to varying concentrations of vitamin D. (**d**) Specificity evaluation of the aptamer-based assay. (**a**–**d**) reproduced from [92] under Creative Commons Attribution License, 2023. (**e**) The monolithically integrated microcantilever includes a microcantilever array, with analog and digital circuit components. (**f**) Schematic of the U-shape (left) and piezoresistive microcantilever sensor (right). (**g**) Results obtained for human IgG measured at varying concentrations. (**h**) The relationship between saturated voltage changes and varying IgG concentrations. (**e**–**h**) reproduced from [93] under Creative Commons Attribution License, 2021. (**i**) The schematic representation of the quarter-configuration Wheatstone bridge. (**j**) Cross-sectional view of PI/Si/SiO_2_ microcantilevers. (**k**) Measurement results for aflatoxin B1 at concentrations of 1, 10, 20, 50 ng/mL. (**l**) Linear correlation of the measured output voltage response and aflatoxin B1 concentrations. (**m**) Detection results for ricin, abrin, and IgG at 100 ng/mL concentrations using microcantilever biosensors functionalized with aflatoxin B1 antibodies. (**i**–**m**) reproduced from [94] under Creative Commons Attribution License, 2021.

Silicon-based microcantilever biosensors are a cutting-edge type of mechanical bio-sensors recognized for their high sensitivity, downsizing potential, and compatibility with sophisticated electronics [91]. These biosensors detect mechanical deflections or changes in resonance frequency caused by biomolecular interactions on the microcantilever surface. Detection is based on two modes: static and dynamic. In the static mode, biomolecule attachment alters surface stress, resulting in cantilever bending, which may be measured by optical or piezoresistive techniques. The cantilever’s resonance frequency alters in the dynamic mode due to changes in mass or stiffness upon target engagement, allowing for high-sensitivity and the label-free detection of proteins, DNA, and microbes in real time [44]. In this regard, Alzahrani et al. proposed a silicon-based microcantilever biosensor for detecting the levels of 25-hydroxyvitamin D (25(OH)D) in the human body [92]. As depicted in Figure 8a, the authors utilized silicon microcantilevers measuring 300 µm in length, 80 µm in width, and 1 µm in thickness. The authors processed the biofunctionalization process by first washing the microcantilevers with piranha solution to remove organic residues, followed by depositing a 5 nm chromium adhesion layer and a 40 nm gold layer using a thermal evaporation system. The gold-coated microcantilevers were immersed in a Bruno aptamer solution (1 × 10^−15^ M) for 3 h at room temperature to immobilize the aptamer on the surface. After incubation, the microcantilevers were washed with phosphate-buffered saline and dried with nitrogen gas to remove unbound aptamers. The successful immobilization was confirmed using contact angle measurements and FTIR spectroscopy, indicating a change in surface wettability and the presence of nucleotide functional groups [92]. This aptamer, known for its high sensitivity and selectivity, allowed the detection of vitamin D through measurable changes in the microcantilever’s deflection and resonance frequency. After the successful immobilization of the aptamer, the microcantilever biosensor exhibited a change in resonance frequency when exposed to increasing concentrations of 25(OH)D ranging from 1 to 300 nM. As shown in Figure 8b, the resonance frequency increased due to the reduction in aptamer mass on the surface which is consistent with the observed deflection behavior. The frequency shift was approximately 5 Hz at 1 nM and 15 Hz at 300 nM with a calculated limit of detection of ~0.3 nM (see Figure 8c), demonstrating the high sensitivity of the biosensor. Moreover, the specificity of biosensor for 25(OH)D was determined through reference experiments with vitamin C and cholesterol by keeping the same concentration of 300 nM for each and a blank solution. Illustrated in Figure 8d, there were no significant changes in microcantilever deflection or frequency, thus confirming the biosensor’s high specificity for 25(OH)D. In another study by Liu et al., a highly sensitive single-chip microcantilever biosensor was developed using silicon-on-insulator CMOS technology for the detection of different biomolecules (human IgG, abrin, and staphylococcus enterotoxin B (SEB)) at high sensitivity [93]. The design integrates a microcantilever array and analog and digital circuits into a unified system as shown in Figure 8e. The array includes twelve rectangular microcantilevers (200 μm × 50 μm × 1 μm) with U-shaped piezoresistors (Figure 8f) which are optimized to 8 kΩ via boron ion implantation. The biosensing system uses three Wheatstone bridges, each consisting of four microcantilevers array (two reference and two measurements, see Figure 8f) to compensate for environmental variations such as humidity and temperature. As depicted in Figure 8f, the reference microcantilevers have a 400 nm buried oxide layer, a 340 nm silicon piezoresistive layer, and a 300 nm SiO_2_ top layer. The measurement microcantilevers include an additional 10/50 nm Ti/Au layer which facilitates accurate and stable detection of surface stress from molecular interactions and is later converted into a differential voltage signal. The output voltage signal can be described as follows:

(8) $$V_{out}=\frac{1}{2}V_{DD}\frac{\Delta R}{R}$$

Here, *V_DD_* represents the supply voltage, *R* denotes the initial resistance of the piezoresistor, and Δ*R* signifies the change in resistance induced by the binding process. Moreover, the deflection sensitivity of the microcantilever is recorded as 0.98 × 10^−6^ nm^−1^ which is given by ΔR/R. Δz^−1^, where ΔR/R is the relative resistance change and Δz^−1^ is the vertical displacement at the free end under force. Before biomolecule detection, the microcantilevers were functionalized with biotinylated goat anti-human IgG to immobilize probes on the Au surface and the sensors were then exposed to human IgG at concentrations of 1.0, 2.0, and 5.7 ng/mL, as illustrated in Figure 8g. Real-time response curves showed a linear relationship between voltage change and IgG concentration depicted in Figure 8h, with responses reaching saturation as more IgG molecules bound to the probes, hence demonstrating the sensors’ repeatability and high sensitivity. The authors also detected abrin (highly toxin protein) and staphylococcus enterotoxin B (SEB, a pathogen) with a calculated LOD of 86 pg/mL and 48 pg/mL, respectively. Also, Tian et al. developed a micro polyimide (PI)/Si/SiO_2_-based flexible piezoresistive microcantilever biosensor for detecting aflatoxin B1, a highly toxic compound commonly found in agricultural products [94]. As shown in Figure 8i, the biosensor schematic illustrates a flexible silicon-based microcantilever with dimensions of 120 μm × 50 μm, fabricated on the single-crystal silicon layer of a silicon-on-insulator wafer. The design integrates a Wheatstone bridge comprising four identical single-crystal silicon piezoresistors: two on the substrate (reference) and two embedded in the microcantilevers (sensing). This configuration provides exceptional mechanical properties, including a spring constant of 21.31 nN/μm and a deflection sensitivity up to 3.54 × 10^−7^nm^−1^. To enhance sensitivity and prevent structural damage, a 1.2 μm thick top polyimide passivation layer with a Young’s modulus of 8.5 GPa was applied to the sensing microcantilever. For specific biomolecule detection, a Cr/Au-modified layer was added to the sensing microcantilever, as depicted in the cross-sectional diagram in Figure 8j, which facilitated the immobilization of biotinylated antibodies on the surface. The detection of aflatoxin B1 was carried out using the biotin–avidin system (BAS). First, streptavidin was attached to the surface of the microcantilevers, followed by treatment with biotinylated aflatoxin B1 antibodies (30 µg/mL). Figure 8k displayed the voltage response for aflatoxin B1 detection at concentrations of 1, 10, 20, and 50 ng/mL. As aflatoxin B1 binds to antibodies on the sensor, it increases surface stress which then leads to higher voltage outputs and longer equilibrium times for higher concentrations. The surface stresses during probe–target interaction cause microcantilever bending and change in resistance and can be expressed as follows:

(9) $$\frac{\Delta R}{R}=(1+2v)\varepsilon +\frac{\Delta \rho }{\rho_{0}}$$

Here, *υ* is the Poisson’s ratio of the piezoresistive material; *ε* is the relative change in the length of the piezoresistor; and Δ*ρ* and *ρ*_0_ represent the change in electrical resistivity and the initial resistivity of the piezoresistor, respectively. The linear correlation between output voltage and aflatoxin B1 concentration (R^2^ > 0.98) is illustrated in Figure 8l with the LOD for aflatoxin B1 is calculated to be 0.1 ng/mL. The biosensor’s specificity shown in Figure 8m, indicates a significantly higher voltage response for aflatoxin B1 compared to similar molecules (e.g., ricin or abrin), confirming the sensor’s selectivity and minimal cross-reactivity. In conclusion, silicon-based microcantilever biosensors represent a highly optimized platform for precise and selective biomolecular detection. Their superior mechanical properties, broad compatibility with various surface functionalization strategies, and capacity for real-time, label-free analysis position them as a valuable tool in clinical diagnostics and biomedical research. The summary of silicon-based biosensors in Table 1 provides a comparative analysis, highlighting their diverse sensing mechanisms and application-specific advantages. FET-based biosensors, particularly SiNW FETs, achieve the lowest LODs, making them ideal for ultra-trace biomolecule detection, while TFET biosensors offer superior sensitivity with minimal power consumption. Optical and photonic biosensors enable high-sensitivity, label-free detection, whereas capacitive and microcantilever biosensors present alternative approaches for molecular diagnostics, each excelling in specificity and continuous monitoring. The choice of biosensor depends on factors such as target analyte, sensitivity requirements, power constraints, and application domain, emphasizing the ongoing need for research to enhance biosensor performance across biomedical platforms.

## 3. Application of Si-Based Platforms in Biosensing Technologies

### 3.1. Miniaturization, Structural Design, and Performance Enhancement

Miniaturization, inspired by Moore’s Law, has significantly influenced the development of Si-based biosensing technologies by enhancing their performance parameters, such as conductivity, sensitivity, and response time. The miniaturization process reduces the dimensions of the sensor components, which leads to an increased surface-to-volume ratio, thereby improving interaction efficiency with biological analytes. Moreover, miniaturized devices allow for integration with microfluidic channels, facilitating the precise and controlled delivery of samples, which enhances detection accuracy in real-time applications [47,95]. Also, the key structural design elements play a pivotal role in optimizing the performance of miniaturized biosensors. Specifically, the integration of nanostructured materials such as nanowires, nanotubes, and nanopillars into silicon platforms enhances sensitivity by increasing the active surface area available for analyte interaction, as detailed in Section 2.1. Silicon nanowires, for instance, benefit from quantum confinement effects that significantly improve their electrical properties, enhancing their ability to detect low-concentration biomarkers [67]. Furthermore, the use of porous silicon serves to augment both surface area and biomolecular interactions, facilitating ultra-sensitive detection capabilities [89]. These advancements have facilitated the development of Si-based biosensors with superior detection capabilities, expanding their applicability in various domains. Moreover, the advancement of fabrication techniques has been integral to addressing challenges related to thermal stability, mechanical integrity, and the integration of flexible substrates for wearable applications. Innovations such as polymer-assisted microfabrication and the incorporation of 2D materials (e.g., graphene) have enabled the production of flexible, stretchable sensors without compromising performance. In wearable biosensors, the development of wireless and energy-efficient platforms has been a major focus, where piezoelectric and solar energy harvesting technologies are incorporated to minimize power consumption, supporting the feasibility of long-term physiological monitoring [50,96,97].

### 3.2. Multifunctionality and Specificity

Si-based platforms are crucial in biosensing technologies due to their multifunctionality and high specificity. The platforms enable the simultaneous detection of multiple biomarkers within a single system, making them highly valuable for real-time health monitoring and rapid diagnosis in clinical applications. The multifunctionality of the platforms allows for the precise detection and quantification of a wide range of biomarkers under various conditions. Additionally, their high specificity enables accurate identification of target biomarkers, offering a significant advantage for precise disease diagnosis and treatment. Recent research has demonstrated various approaches to enhance these characteristics.

Multifunctionality is a defining feature of advanced Si-based biosensing platforms, enabling the simultaneous detection of multiple biomarkers within a single device. Achieving this requires sophisticated sensor designs that leverage nanomaterials and data fusion algorithms. For example, graphene field-effect transistor (GFET)-based sensors have been enhanced with specific nanoclusters to enable the detection of biomarkers such as dopamine and glucose concurrently, achieving high accuracy and sensitivity in complex biological samples [38]. A data fusion algorithm was used to achieve high sensitivity and accuracy, addressing conditions like schizophrenia. Similarly, silicon nanowires integrated into PDMS microfluidic systems facilitate the simultaneous detection of α-fetoprotein (AFP) and carcinoembryonic antigen (CEA), which are indicative of primary hepatic carcinoma, thus enabling early-stage cancer diagnosis through highly sensitive serum analysis [98]. These multifunctional platforms are typically designed using multi-layer architectures or hybrid materials, where each layer or material is selected to capture specific biomolecules, allowing for multiplexed detection [99]. Additionally, microfluidic channels are often incorporated into the sensor design to efficiently manage sample flow and reduce the volume of reagents required, thereby enhancing sensor throughput and analytical performance [30]. These advancements in Si-based platforms highlight their potential to improve disease diagnostic applications by enabling simultaneous detection of multiple biomarkers.

Specificity is a crucial parameter in biosensors for accurately detecting biomarkers in complex biological matrices. Recent advancements in material science and surface chemistry have substantially enhanced the specificity of Si-based platforms. Techniques such as aptamer- or antibody-based functionalization, as well as molecularly imprinted polymers (MIPs), are employed to selectively capture target biomarkers, minimizing nonspecific binding events [100]. Furthermore, surface modifications using titanium dioxide (TiO_2_) or carbon-based nanomaterials (e.g., graphene) improve both the stability and selectivity of sensors by providing biocompatible, non-fouling surfaces that resist interference from non-target species. For instance, Ryazantsev et al. improved the detection of troponin I by functionalizing Ta_2_O_5_-gated CMOS ISFETs with aptamers, achieving a detection limit of 3.27 ng/mL, critical for cardiac biomarker detection [101]. Similarly, Kuo et al. utilized a CMOS capacitive nanobiosensor for microRNA-195 detection, reducing nonspecific interactions and achieving an ultra-sensitive detection limit of 0.617 fM, which is crucial for early cancer diagnostics [102]. Also, advances in signal transduction have significantly improved the specificity and sensitivity of silicon-based biosensors. FETs, carbon nanotubes (CNTs), and gold nanoparticles (AuNPs) are commonly used as signal amplification elements in these sensors to enhance detection sensitivity [103,104]. The use of plasmonic effects in combination with nanomaterials allows for highly sensitive detection of biomolecular interactions, while maintaining specificity even in the presence of complex biological backgrounds [105]. These advances allow silicon-based biosensors to achieve exceptional detection performance, making them suitable for a variety of biomedical applications

Furthermore, the design of silicon biosensors for targeted applications necessitates customization to optimize performance for specific biomarkers. For example, sensors for small molecule detection (e.g., glucose sensors) require designs that prioritize high specificity for low molecular weight targets, while protein sensors benefit from increased surface area for large biomolecule capture. Multiplexed detection of biomarkers in clinical diagnostics requires array-based sensor designs, where each sensor element is functionalized with different receptors, allowing simultaneous measurement of multiple analytes in parallel [106]. Hybrid sensor architectures, where Si-based transistors are coupled with microfluidic platforms, enable the integration of multiple detection modes and allow for enhanced biomarker recognition in complex samples [107].

While these advances in multifunctionality and specificity offer significant potential for silicon biosensor platforms, challenges remain in wearable and implantable devices, including biocompatibility, mechanical fragility, miniaturization, and energy supply constraints. Ongoing research is focused on overcoming these limitations by improving material integration, sensor robustness, and low-power consumption [108]. These topics will be further explored in the following section.

## 4. Challenges, Limitations, and Advances in Si-Based Biosensors for Wearable Applications

Silicon-based biosensors have demonstrated significant potential for wearable applications; however, several challenges and limitations hinder their widespread adoption. These include fragility and rigidity, challenges in biomolecule interactions, miniaturization constraints, signal interference issues, and power supply limitations. Overcoming these challenges requires advancements in materials science, fabrication techniques, and integration strategies with emerging technologies, particularly artificial intelligence and machine learning.

### 4.1. Challenges and Limitations

Fragility is a major concern in silicon biosensors applications that require durability and resilience. Si-based biosensors are vulnerable to physical damage, such as cracking or chipping, during handling, transport, or even during operation in harsh environments. Recently, hydrogel-based wearable electrochemical biosensors (HWEBs) are emerging as a promising alternative to traditional analytical methods. These sensors utilize hydrogel materials as platforms for immobilizing biorecognition elements, offering high selectivity and sensitivity [109]. Integrating physical, chemical, or electro-physiological sensors into wearable and implantable devices provides a reliable way to continuously monitor physiological parameters, enabling the collection of accurate data that can help to minimize treatment complexity [110]. However, the fragile and rigid properties of Si-based biosensors limit their application, especially in scenarios that require deformation, bending, or handling under dynamic conditions. A promising solution involves incorporating flexible substrates, such as polydimethylsiloxane (PDMS) and polyethylene terephthalate (PET), which enhance mechanical durability and improve adaptability for wearable application [111].

Another significant challenge is ensuring stable and reliable biomolecular interactions in silicon biosensors designed for long-term monitoring of physiological parameters. Effective biosensors should have the ability for in situ, real-time, and simultaneous multi-marker detection, while being disposable, flexible, small in size, and low-cost to fabricate [112]. Although Si-based biosensors are gaining popularity because of their multifunctional features, the biophysical interactions with the Si interface remain inadequately explored [113]. Efficiently functionalizing the silicon surface with biomolecules (e.g., antibodies, enzymes, or nucleic acids) is crucial for biosensor performance. However, the silicon surface is often chemically inert and requires complicated surface treatments to introduce active groups or to bind biological molecules. For example, Hansel et al. illustrated that when porous silicon nanostructures interface with cells, the formation and maturation of focal adhesions (FAs) at the cell–material interface are inhibited, which leads to reduced cytoskeletal tension and lessened activity of mechanoresponsive transcriptional regulators [114]. It is challenging to maintain stable, reproducible functionalization with high specificity. Variations in surface chemistry can affect sensor performance, causing issues like nonspecific binding or biofouling that reduce sensitivity and selectivity. However, recent advances in nanostructuring, including the use of porous silicon nanostructures, have enhanced biomolecular adhesion and reduced biofouling, improving sensor performance.

Miniaturization is another key limitation. The fabrication of silicon biosensors often involves expensive materials and advanced manufacturing processes, leading to devices that are frequently bulky and inefficient. Challenges in automating and miniaturizing conventional designs for multifunctional applications further escalate costs and hinder scalability. Incorporating microfluidics into biosensors, while advantageous for miniaturization and improved reaction control, adds another layer of complexity. This integration requires precise engineering, significantly increasing production costs. For instance, SiNW-FETs are highly sensitive but particularly challenging to fabricate. The “top-down” approach, commonly used for their production, relies on advanced lithography and nanofabrication techniques, which are both expensive and technologically demanding [115]. To address this, researchers have developed scalable manufacturing techniques, including inkjet printing and roll-to-roll fabrication, to produce cost-effective and compact biosensing platforms [116].

Signal interference remains a major concern in silicon biosensors as they are highly sensitive to environmental factors such as temperature, pH, ionic strength, and interfering substances. However, variations in these conditions can lead to noise, significantly lowering the signal-to-noise ratio (SNR) and making it difficult to accurately read the current signals in wearable sensors. This limitation can lead to challenges to detect small signals from analytes such as saliva and bodily fluids. In silicon biosensors employing CMOS technology, noise sources such as thermal noise, flicker noise, and shot noise can reduce performance at the interface. Thermal noise is caused by the thermal motion of the feedback resistance and the sensor output resistance. Flicker noise, which mainly appears in the low-frequency range, depends on factors such as the oxide layer characteristics and the surface state of the MOSFET. Shot noise, although negligible in CMOS, arises due to the discrete movement of current in input devices. Increasing the feedback resistance can enhance the sensor’s ability to detect small current flows, but this can also increase thermal noise, reducing the signal-to-noise ratio (SNR) and potentially lowering the sensor’s sensitivity [117,118]. To mitigate these issues, organic field-effect transistors (OFETs) have been employed as alternatives to Si-FET sensors. In this regard, Wei Tang et al. designed an OFET sensor to overcome the noise limitations of conventional Si-FET sensors and improve the SNR for miRNA detection. By reducing the trap state density and integrating a high-k/low-k structure gate dielectric, the OFET sensor achieved a superior SNR and lower noise compared to traditional Si-FET sensors. These improvements resulted in enhanced detection limits, operational voltage, and static power [119].

Power supply constraints further limit the viability of Si-based biosensors for continuous bio-detection. To read small bio-signals in the range of microvolts to millivolts in wearable sensors, it is essential to minimize power consumption and ensure continuous power supply [120,121,122]. However, silicon biosensors face significant challenges in power supply due to static and dynamic power consumption. Overcoming these challenges is critical for integrating silicon biosensors into wearable sensors. For instance, silicon-based sensors utilizing CMOS technology experience static power consumption caused by leakage currents, which become more significant as miniaturization progresses for biosensing purposes. Additionally, dynamic power consumption occurs during the charging and discharging of load capacitances due to circuit switching operations [123]. To overcome this challenge, energy-harvesting techniques such as piezoelectric, triboelectric, and biofuel cell-based systems have been integrated into silicon biosensors, enabling self-powered operation and reducing dependency on external power sources [124].

### 4.2. Advancements and Integration into Wearable Technologies

Despite these challenges, significant advancements have been made in the integration of Si-based biosensors with wearable technologies and AI. Recent studies have demonstrated flexible Si-based biosensors capable of withstanding mechanical deformations while maintaining high sensitivity and specificity [125,126]. For instance, Seyong Oh et al. developed a flexible synaptic device based on a silicon–indium–zinc-oxide (SIZO)/ion gel hybrid structure, which exhibited exceptional durability and stability after extensive bending cycles [127]. Such innovations are crucial for enabling real-time, continuous health monitoring. The integration of AI and ML algorithms has further enhanced the capabilities of silicon-based wearable biosensors. AI-driven data processing allows for real-time interpretation of biosensor signals, reducing noise and improving diagnostic accuracy. In particular, ML models trained on large datasets can identify patterns and correlations within biosensor data, facilitating early disease detection and personalized health monitoring [128,129]. Neuromorphic computing approaches have also been explored to mimic biological signal processing, improving response times and computational efficiency in wearable biosensing applications [130]. Additionally, researchers are investigating hybrid biosensing platforms that combine Si-based sensors with emerging materials such as graphene, carbon nanotubes, and transition metal dichalcogenides (TMDs) [131]. These materials offer superior mechanical flexibility, enhanced electrical conductivity, and improved biocompatibility, paving the way for next-generation silicon-based wearable biosensors.

## 5. Conclusions

In conclusion, this review underscores the pivotal role of silicon-based biosensors in advancing biomedical applications over the past decade. Silicon’s exceptional properties, including high sensitivity, superior thermal and chemical stability, low power consumption, and enhanced signal-to-noise ratio, render it an indispensable material for biosensing technologies. Its tunable electrical conductivity, achieved through impurity doping, has further expanded its utility, enabling the development of innovative nanostructures such as nanowires and nanosheets for sensor prototypes. These advancements enhance the performance of silicon biosensors and position them as fundamental building blocks for the next generation of multifunctional and highly precise biosensing systems.

The integration of silicon-based biosensors into multifunctional platforms holds promise for reducing fabrication costs and minimizing electronic waste, thereby improving their scalability and environmental sustainability. Continued progress in silicon microfabrication techniques and material engineering is expected to drive the miniaturization and dynamic adaptability of biosensors, which are critical for portable, wearable, and point-of-care medical devices. Furthermore, the hybridization of silicon with complementary materials, such as nanoparticles or organic polymers, may lead to the development of composite biosensors with unprecedented sensitivity and specificity for detecting subtle biological changes.

While challenges remain, particularly in addressing silicon’s intrinsic rigidity and brittleness for wearable applications, advancements in surface modification, flexible substrate integration, and nanotechnology are anticipated to overcome these limitations. Such innovations will pave the way for the realization of robust, cost-effective, and scalable point-of-care silicon biosensors capable of real-time, on-site diagnostics. As the intersection of silicon technology and artificial intelligence continues to evolve, the synergy between these domains will likely revolutionize biosensing systems, enabling predictive and personalized healthcare solutions for the future.

## Figures and Tables

**Table 1:** Summary of the reported Si-based biosensors.

Si-Based Biosensor Type	Bioreceptor	Target	Sensitivity	LOD	Specificity	Ref
SiNWs FET	antibody	Ag85B biomarker	5.96 nA/log (fg/mL)	0.01 fg/mL	High	[65]
	antibody	293F exosome	3.80 × 10^4^ to 3.80 × 10^9^ particles/mL	1078 particle/mL	High	[66]
	antibody	Cystatin C biomarker	0.42 V/dec	0.2529 ag/mL	High	[67]
ISFET	antibody	cTnI	132 pA	0.3 pg/mL	High	[70]
	antibody	HSA	High	5 μg/mL	High	[71]
	polymeric membrane	Na^+^	60.7 ± 0.5 mV	9.4 × 10^−6^ M	High	[72]
Hydrogel-gated FET	hydrogel	SARS-CoV-2	30 mV ± 5.7 mV	1 ng/mL	-	[75]
	hydrogel	pH sensing	100 mV/pH	-	-	[76]
TFET	nanocavity	biomolecules	1193	-	High	[81]
	nanocavity	biomolecules	High	-	High	[57]
Capacitive	lectin	PSMA	2.64 nF/M	10 pM	High	[83]
	antibody	CRP	High	1 pg mL^−1^	High	[84]
	SAM	ssDNA	0.061 fg∙Hz^−1^∙µm^2^	0.635 µmol∙L^−1^	High	[85]
Optical	antibody	myoglobin	536 nm/RIU	35.7 ng/mL	-	[88]
	lectin	*E. coli*/*S. aureus*	3 × 10^3^ to 3 × 10^5^ cells/mL	103 cells mL^−1^	High	[89]
Photonic	antibody	CRP/*E. coli*	50.8 pm/μg/mL 42 pm	0.29 μg/mL10 cells/mL	High	[90]
Microcantilever	aptamer	25(OH)D	5 Hz at 0.001 µM	~0.3 nM	High	[92]
	antibody	IgG, abrin, SEB	0.98 × 10^−6^ nm^−1^	48 pg/mL	High	[93]
	antibody	aflatoxin B1	3.54 × 10^−7^ nm^−1^	0.1 ng/mL	High	[94]
